# Influence of different stocking density on the growth, feed efficiency, and survival of Majalaya common carp ( *Cyprinus carpio* Linnaeus 1758)

**DOI:** 10.12688/f1000research.16875.1

**Published:** 2018-12-10

**Authors:** Mir'atul Hayat, Rudy Agung Nugroho, Retno Aryani

**Affiliations:** 1 Animal Physiology, Development, and Molecular Laboratory, Department of Biology, Mulawarman University, Samarinda, East Kalimantan, 765123, Indonesia; 2 Animal Anatomy and Microtechnique Laboratory, Department of Biology, Mulawarman University, Samarinda, East kalimantan, 765123, Indonesia

**Keywords:** Majalaya Common carp, stocking density, growth

## Abstract

**Background: **Stocking density is key to successful Majalaya common carp ( *Cyprinus carpio* Linnaeus 1758) culture which is a valuable fish culture in Indonesia.  The aim of the present study was to evaluate the growth statues, feed utilization, and survival rate of Majalaya common carp (reared with different stocking density.

**Methods: **In total, 1400 fish were randomly distributed into four replicates of four different groups of stocking density: 50, 75, 100, and 125 fish m ^−3^. All fish were fed using a satiation method, three times per day with commercial diet for 12 weeks. At the end of the trial week, growth, feed utilization, and survival were determined. Water quality measures, such as dissolved oxygen (mg L ^-1^), temperature (°C), pH, NH ^3^ (mg L ^-1^), and NO _2_ (mg L ^-1^) were also measured once a week during the trial.

**Results: **Similar weight gain and SGR were found in Majalaya common carp reared at stocking densities of 50 to 100 fish m ^3^. However, 125 fish m ^-3^ density may reduce weight gain and SGR. The average weekly and daily weight gain of Majalaya common carp significantly increased when reared from 50 to 100 fish m ^-3^ and remained constant at 125 fish m ^-3^ density. Meanwhile, feed conversion ratio and survival of Majalaya common carp were not affected by any stocking density.

**Conclusions:** A stocking density of 100 fish m ^-3^ exhibited significantly higher growth of Majalaya common carp in hapa net ponds among the treatment. Temperature ranges of 29.20-33.38°C, pH 7.47-8.22, DO 4.76-7.55 (mg L ^-1^), NH _3_ 0-0.5 mg L ^-1^, and NO _2_ 0-1 mg L ^-1 ^were found to provide optimum condition to the fish.

## Introduction

One of the important factors related to fish culture productivity is stocking density ^1– 4^. Past research has found that growth, feed efficiency and survival can be optimized by considering stocking density in fish culture operations ^5– 7^. Besides stocking density, water quality is another factor that must be taken into consideration. Water quality is associated with stocking density in term of the availability of food and condition of the environment in fish culture ^8^. Breeding of the Majalaya common carp ( *Cyprinus carpio* Linnaeus 1758) is the result of selection conducted in Indonesia ^9^. The Majalaya carp belongs to the Cyprinidae family and is an important fish to be cultured in Indonesia ^10^. Though several research studies regarding stocking density in some fish have been conducted ^2, 3, 11– 13^, the influence of different stocking densities on the growth, feed efficiency, and survival of the Majalaya common carp in hapa fish ponds has never been determined. Thus, the purpose of the research was to evaluate the growth statues, feed efficiency, and survival rate of the Majalaya common carp, reared at different socking density, viz: 50, 75, 100, and 125 fish m ^−3^ in the hapa fish pond.

## Methods

### Carp culture conditions

In total, 1400 Majalaya common carp (mean initial weight ±26.22 g, random sex) were distributed into four groups with four replications each groups and reared with different stocking densities: 50, 75, 100, and 125 fish m ^−3^ in hapa fish ponds (1 x 1 x 1.2 m) for 12 weeks. During the trial, all fish were fed with a commercially available diet (PT Japfa Comfeed, No. reg. KKP RI IN 682072012, containing 30% protein, 5.5% fat, and 5% fibre). All fish were fed to satiation three times per day. At the end of the trial, growth parameters for each overall hapa fish pond, such as final weight, weight gain, average weekly weight gain (AWG), daily weight gain (DWG), specific growth rate (SGR), feed conversion ratio (FCR) were determined using an equation previously described by Abdel-Tawwab *et al*. ^14^, Muchlisin *et al*. ^15^, Tran-Ngoc *et al*., ^16^ Asriqah *et al*. ^17^. Meanwhile, survival was calculated with equation as used by Nugroho *et al*. ^18^.

### Measuring water quality

Water quality, such as dissolved oxygen (DO) (mg L ^-1^) and temperature (°C) were assessed using a digital water checker (YSI™ Model 550A DO Meter; Fisher Scientific, USA). The pH was measured with a pH-meter (CyberScan pH 11; EuTech Instruments, Singapore), while NH _3_ (mg L ^-1^), and NO _2_ (mg L ^-1^) were detected using a Sera test kit (Sera GmbH D52518, Heinsberg, Germany). All water quality parameter were measured once a week during the trial.

### Statistical analysis

Data were analysed using SPSS 22 (SPSS, Inc., USA). Growth, FCR, and survival were subjected to analysis of variance, followed by Duncan post hoc to evaluate significant differences among the groups. Water quality was descriptively analysed. All significant tests were at *P*<0.05.

## Results

### Effect of stocking density

Present study showed that stocking density from 50 to 100 fish m ^3^ of Majalaya common carp in the hapa fish pond resulted in similar weight gain and SGR. However, stocking density higher than 100 fish m ^-3^ may reduce weight gain and SGR. The AWG and DWG of Majalaya common carp showed a significantly increase trend when reared from 50 to 100 fish m ^-3^ and remained constant at 125 fish m ^-3^ density. Meanwhile, FCR and survival were not affected by any stocking density ( Table 1; raw data available on OSF ^19^). The high density (100 fish m ^−3^) could be more profitable for the Majalaya common carp farms in Indonesia in terms of reduced land cost and facilities.

**Table 1.** Mean ± standard error of growth statues and feed utilization of *Cyprinus carpio* Linnaeus 1758 Majalaya reared with different stocking density for 12 weeks in hapa fish ponds.

Parameters	Stocking density (fish m ^-3^)			
	50	75	100	125
Initial weight (g)	1310±0 ^a^	1970±0 ^b^	2620±0 ^c^	3280±0 ^d^
Final weight (g)	6205.00± 76.84 ^a^	8467.50±347.16 ^b^	11315.00±324.17 ^c^	12892.50±669.19 ^d^
Weight gain (g)	99.19 ±3.98 ^a^	87.03±4.77 ^ab^	88.39±3.35 ^ab^	79.43±5.43 ^b^
AWG (g week ^-1^)	408.25±14.73 ^a^	541.50±28.99 ^b^	724.75±26.92 ^c^	801.00±55.87 ^c^
DWG (g day ^-1^)	58.25±2.28 ^a^	77.25±3.94 ^b^	103.50±3.96 ^c^	114.25±8.09 ^c^
SGR (% day ^-1^)	1.86±0.035 ^a^	1.73±0.052 ^ab^	1.75±0.034 ^ab^	1.65±0.061 ^b^
FCR	0.66±0.019 ^a^	0.66±0.010 ^a^	0.68±0.020 ^a^	0.70±0.026 ^a^
Survival (%)	99.00±0.57 ^a^	99.66±0.33 ^a^	98.75±0.25 ^a^	97.60±0.65 ^a^

Different superscript letters ( ^a, b, c, d^) indicate significantly different means for different group of diets at *P*<0.05. Initial and final weights are the biomass weights.

### Effect of density on water parameters

Water parameters showed a suitable condition for culturing Majalaya common carp at different stocking density up to 125 fish m ^-3^. The temperature ranged 29.20–33.38°C, pH range of 7.47–8.22, DO of 4.76–7.55 mg L ^-1^, NH _3_ 0–0.5 mg L ^-1^, and NO _2_ 0–1 mg L ^-1^, respectively ( Table 2). The data showing the growth parameters and water quality parameters can be seen on OSF ^19^.

**Table 2.** Mean ± standard error of water quality during the stocking density trial of Majalaya Common carp reared in hapa fish ponds for 12 weeks.

Parameters	Stocking density (fish m ^-3^)				Value	
	50	75	100	125	Minimum	Maximum
DO (mg L ^-1^)	6.07±0.13	6.07±0.13	6.07±0.13	6.07±0.13	4.76	7.55
Temperature (°C)	30.38± 0.16	30.38±0.16	30.38±0.16	30.38±0.16	29.20	33.80
pH	7.84 ±0.02	7.84 ±0.02	7.84 ±0.02	7.84 ±0.02	7.47	8.22
NH _3_ (mg L ^-1^)	0.08±0.02	0.08±0.02	0.08±0.02	0.08±0.02	0	0.50
NO _2_ (mg L ^-1^)	0.12±0.04	0.12±0.04	0.12±0.04	0.12±0.04	0	1.00

## Discussion

Previous research indicated that high growth rates, high levels of survival and better FCR may be due to low feed competition and density ^2, 20, 21^. The present study stated that a stocking density up to 100 fish m ^-3^ resulted in similar weight gain and SGR, but this was reduced at the highest density (125 fish m ^-3^). Meanwhile, FCR and survival were not affected by any stocking density. This finding is similar to previous research that survival and growth of fish were independent of the stocking density ^22, 23^. In addition, the growth and survival of fish in practical culture may also depend on the species. For example, the survival and growth rate of the catfish *Rita rita*, at different densities of 10, 20 and 30 fish per cistern, resulted in the highest survival and SGR in 20 fish per cistern. Further, no competition for feed and space observed at low density culture of this fish ^24^. In contrast, a prior study revealed that survival rate in aquatic animals was negatively correlated with stocking which could be due to high competition and space for the fish ^2^.

Excess feed remaining in the pond, as well as stocking density, might change the water quality. In this research, the water quality parameters during the trials showed no effects on the growth and survival of fish culture during the trial. The present findings are concomitant with those of previous studies, which found that water quality measures such as temperature, DO, pH, NO _2_ and NH _3_ measured in similar current experimental setups are all within the acceptable value for culturing fin fish in tropical regions ^25, 26^. The data regarding the growth status and water quality mean, minimum and maximum values can be obtained in Dataset 1.

## Conclusion

The Majalaya common carp can be reared at stocking density up to 100 fish m ^-3^ without negative effects on the growth, FCR, and survival. The water quality is suitable condition and suggested for culturing Majalaya common carp in hapa fish ponds. Further research needs to be conducted to evaluate the fillet and carcass proximate composition, and immune system of Majalaya common carp when reared at high stocking density.

## Data availability

Raw data for Tables can be accessed on OSF, DOI: https://doi.org/10.17605/OSF.IO/TGC45 ^19^.

Data are available under the terms of the Creative Commons Zero "No rights reserved" data waiver (CC0 1.0 Public domain dedication).
