# Supplementary material for: Influence of different stocking density on the growth, feed efficiency, and survival of Majalaya common carp ( Cyprinus carpio Linnaeus 1758)
Source: F1000Res. 2018 Dec 10;7:1917. [Version 1] doi: 10.12688/f1000research.16875.1 (PMC6338240; doi:10.12688/f1000research.16875.1)
Supplement: Peer review history

# Peer review: Referee response for version 1

**DOI:** 10.5256/f1000research.18450.r41743

**Published:** 2019-01-17

**Authors:** Kim T. Tran-Ngoc

**Affiliations:** 1 Faculty of Fisheries, Nong Lam University, Ho Chi Minh City, Vietnam

The experimental design and procedures sound good overall. The findings are obtained for better understanding of Majalaya common carp cultured with different stocking density. 

 I have some comments that may improve your work:

1. The information about the feed is lacking: pellet size? Extruded pellets?
2. In Table 1: the initial weight and final weight can be confused between biomass or individual fish. Please add details for this. 
3. In Table 1: please add the explanation for AWG, DWG, SGR and FCR in the footnote. The table and footnote should be understandable without distracting the reader from the main text.

Additional comments:

1. **Comment 1**: Table 1 shows that the final biomass weight was significantly different between treatments. You claim it was because of stocking density. From my view, since the initial biomass weight is already significantly different, that can be a main reason that lead to a difference in final weight. Then, either stocking density or initial weight has an effect on final weight. Could you please explain more about this?
2. **Comment 2**: In the discussion part “this finding is similar to previous research that survival and growth of fish were independent of the stocking density” that from of my view, it is much too subjective. You refer to 2 papers to support your findings.
  However, growth, survival and yield effects of stocking density on aquaculture are well known for a diversity of species and seem to impact production differently. Both growth performance and survival rate, for instance, tend to be higher in low stocking density in the African catfish, *C. gariepinus* (Hecht *et al.*,1996 ^1^), *Oreochromis* spp. (Sorphea *et al.*, 2010 ^2^) and Thai climbing perch *Anabas testudineus* (Khatune-Jannat *et al.*, 2012 ^3^). Therefore, I suggest you should paraphrase this sentence.
3. **Comment 3**: The feeding was ad libitum in your method. Do you have excess feed remaining in the hapa? If yes, how do you manage it? How is feed intake calculated in this experiment? Does stocking density affect feed intake?

I have read this submission. I believe that I have an appropriate level of expertise to confirm that it is of an acceptable scientific standard.

## References

[1] HechtTOellermannLVerheustL: Perspectives on clariid catfish culture in Africa. *Aquatic Living Resources*.1996;9: 10.1051/alr:1996054 197-206 10.1051/alr:1996054

[2] SorpheaSLundhTPrestonTRBorinK: Effect of stocking densities and feed supplements on the growth performance of tilapia (Oreochromis spp.) raised in ponds and in the paddy field. *Livestock Research for Rural Development*.2010;22(11) : Reference source

[3] Khatune-JannatMRahmanMMBasharMAHasanMNAhamedF: Effects of Stocking Density on Survival, Growth and Production of Thai Climbing Perch (Anabas testudineus) under Fed Ponds. *Sains Malaysiana*.2012;41(10) :1205-1210 Reference source

# Peer review: Referee response for version 1

**DOI:** 10.5256/f1000research.18450.r41741

**Published:** 2019-01-10

**Authors:** Zainal Abidin Muchlisin

**Affiliations:** 1 Department of Aquaculture, Faculty of Marine and Fisheries, Syiah Kuala University, Banda Aceh, Indonesia

- Title: Sufficient.
- Abstract: Sufficient.
- Keywords: I think it would be better if the keywords are different from the words which already exist in the title.

Introduction:

- The introduction should be started with the general information about the culture of Majalaya common carp *Cyprinus carpio* in Indonesia and the world, not directly with the problem statement. What are the advantages of this species, etc.?
- This species is known as common carp, but in this study the authors add the “Majalaya” common carp as a common name, so the authors have to introduce why the *Cyprinus carpio* is called Majalaya common carp; maybe this is a new strain or variety which resulted from cross-breeding between species X and species Y.

Methods:

- Please clarify where the experimental fish come from - whether they come from the wild or from a hatchery. If from a hatchery, from which location? Etc.
- Before distribution into the hapas, did you acclimatise the experimental fish? If yes, for how long and what feed were they fed on during acclimatisation?
- I suggest deleting the producer name of the feed; just say “commercial diet”.
- You fed the fish three times a day, at what times exactly?
- How many times was the weight gain measured? For example 2 weeks interval for 12 weeks? Etc.

Results:

- Please add information about the results of the ANOVA test, whether the treatment gave the significant effect or not to the measured parameters.
- The FCR data are lower than 1 in all treatments; it means that to get 1 kg of fish we need 0.7 kg of feed. Are you sure about that? I think it is impossible except you used the additional feed for example plankton, or the plankton were available without attention, where the biomass of these plankton was not included in the calculation. Please clarify.

Discussion:

- Extend the discussion by comparing with other studies; maybe some studies are in agreement with your findings and some previous studies are contradictory, so that we have discussed both phenomena.

Conclusion:

- You said that “The Majalaya common carp can be reared at stocking density up to 100 fish m ^-3^ without negative effects on the growth” - be careful, can be reared until what size?

I have read this submission. I believe that I have an appropriate level of expertise to confirm that it is of an acceptable scientific standard.
